# Explicit Instructions Increase Cognitive Costs of Deception in Predictable Social Context

**DOI:** 10.3389/fpsyg.2015.01863

**Published:** 2015-12-15

**Authors:** Marcel Falkiewicz, Justyna Sarzyńska, Justyna Babula, Iwona Szatkowska, Anna Grabowska, Edward Nęcka

**Affiliations:** ^1^Departament of Neurophysiology, Nencki Institute of Experimental Biology, Warsaw, Poland; ^2^University of Social Sciences and Humanities, Warsaw, Poland; ^3^Jagiellonian University, Cracow, Poland

**Keywords:** deception, lying, cognitive load, stereotype, schema, social cognition

## Abstract

Convincing participants to deceive remains one of the biggest and most important challenges of laboratory-based deception research. The simplest and most prevalent method involves explicitly instructing participants to lie or tell the truth before presenting each task item. The usual finding of such experiments is increased cognitive load associated with deceptive responses, explained by necessity to inhibit default and automatic honest responses. However, explicit instructions are usually coupled with the absence of social context in the experimental task. Context plays a key role in social cognition by activating prior knowledge, which facilitates behaviors consistent with the latter. We hypothesized that in the presence of social context, both honest and deceptive responses can be produced on the basis of prior knowledge, without reliance on truth and without additional cognitive load during deceptive responses. In order to test the hypothesis, we have developed Speed-Dating Task (SDT), which is based on a real-life social event. In SDT, participants respond both honestly and deceptively to questions in order to appear similar to each of the dates. The dates are predictable and represent well-known categories (i.e., atheist or conservative). In one condition participants rely on explicit instructions preceding each question (external cue). In the second condition no explicit instructions are present, so the participants need to adapt based on prior knowledge about the category the dates belong to (internal cue). With internal cues, reaction times (RTs) are similar for both honest and deceptive responses. However, in the presence of external cues (EC), RTs are longer for deceptive than honest responses, suggesting that deceptive responses are associated with increased cognitive load. Compared to internal cues, deception costs were higher when EC were present. However, the effect was limited to the first part of the experiment, only partially confirming our initial hypothesis. The results suggest that the presence of social context in deception tasks might have a significant influence on cognitive processes associated with deception.

## Introduction

One of the most difficult aspects of scientific research on deception is the ecological validity of tasks used in the experiments. Apart from a few studies using paradigms with excellent ecological validity (Baumgartner et al., 2009; Greene and Paxton, 2009; Sip et al., 2010, 2012; Abe and Greene, 2014), a large chunk of contemporary research on cognitive and neural correlates of deception is based on Differentiation-of-Deception (DOD) Paradigm (Furedy et al., 1988) and it’s variant known as Sheffield Lie Test—SLT (Spence et al., 2001). SLT consists of a set of questions, usually related to the episodic (“were you in China for your last holiday?”) and semantic (“Is Paris the capital of Italy?”) memory of the participant. Every question is preceded with a cue, which indicates what type of response is expected for the following question. The same question is usually repeated twice, one with “truth” and the other with a “lie” cue. Therefore, each question provides within-subject control for itself, eliminating the effect of possible confounders on measures such as reaction times (RTs), skin conductance and brain activity.

Despite serious doubts about the face validity of instructed deception paradigms (Sip et al., 2008; Kanwisher, 2009), its variants have been and still are widely used in behavioral and neuroimaging studies on deception (Sheridan and Flowers, 2010; Kaylor-Hughes et al., 2011; Verschuere et al., 2011, 2012; Debey et al., 2012; Ito et al., 2012; Marchewka et al., 2012; Jiang et al., 2013; Vartanian et al., 2013). The results of these studies are largely consistent, most often reporting longer RTs for “lie” compared to “truth” trials and increased brain activity in regions related to cognitive control, indicating that additional cognitive load is associated with deception compared to truth-telling (Christ et al., 2009; Abe, 2011; Gamer, 2014; Lisofsky et al., 2014). However, it still remains unknown how reliable these processes are across different deception contexts (Farah et al., 2014). Given the complexity and variety of forms in which deception occurs in real life, one might suspect that cognitive processes associated with instructed lying cannot be generalized to deception *per se*, but instead are specific to the context in which they are produced. Therefore, further studies are needed to address this issue. In this paper we propose a new experimental method of studying deception, which allows to incorporate elements of social context into a low-stakes deception context. We argue that the presence of explicit cues might increase the cognitive load associated with deceptive (compared to honest) responding when social context is present. In order to present our argument, we must first consider the role of prior social knowledge in social interactions.

Humans accumulate knowledge through detection of regularities in events they experience through life. At any point in time, prior knowledge is used to guide behavior, make predictions, and facilitate the acquisition of new knowledge. Experiments have shown that activation of prior knowledge facilitates remembering words or facts congruent with that knowledge (Meyer and Schvaneveldt, 1971; Stangor and McMillan, 1992; Brod et al., 2013). If prior knowledge has been activated, it is difficult to consciously avoid utilizing it (Marsh et al., 1999). The same principles apply to social interactions. During social interactions prior knowledge related to similar situations and their participants is used to fulfill expectations, behave appropriately, or initiate actions with the intention to achieve a particular goal. Prior knowledge can take many forms, including stereotypes or schemas. These forms can be activated by simple cues like skin color (Macrae and Bodenhausen, 2000), a prime associated with a schema (Wittenbrink et al., 1997), or an explicit verbal label (Devine, 1989). In most cases the knowledge activation is automatic and might be unconscious (Allport, 1954; Devine, 1989; Bargh et al., 1996; Hilton and Von Hippel, 1996; Macrae et al., 1997; Wyer, 2013). It has been demonstrated that black people are more likely than whites to get shot in a Police Officer’s Dilemma task (Correll et al., 2002, 2006), most likely due to activation of a stereotype of an aggressive black person. A less extreme example of the influence of stereotype activation comes from a study where participants with activated rudeness stereotype were more likely to interrupt the experimenter (Bargh et al., 1996). Although stereotyping and categorization have negative connotations and in most cases have been studied as such, it allows reduction of uncertainty about the environment (Hogg, 2000). Taken together, these studies demonstrate that behaviors congruent with activated prior knowledge are facilitated.

Experimental deception tasks usually do not provide any familiar social context for the subjects. Thus, no prior knowledge can be activated and subjects cannot predict what they will be asked about and what the purpose of these questions is. The decisions to respond honestly or deceptively are based solely on cues preceding each question. Since no prior expectations are active in working memory, the only way deceptive responses can be emitted is by negation of truth. Negation produces additional cognitive load as repeatedly reported in instructed lying paradigms, consistent with theories claiming that deception is usually more cognitively demanding than truth-telling (Spence et al., 2001; Walczyk et al., 2005, 2009; Vrij et al., 2008; Sheridan and Flowers, 2010; Verschuere et al., 2010, 2011; Kaylor-Hughes et al., 2011; Debey et al., 2012; Marchewka et al., 2012). This particular process of generating deceptive responses is accurately described by ADCM model (Walczyk et al., 2005).

We argue that if the subjects were able to use prior social knowledge in the experimental situation, behaviors consistent with the prior knowledge would be facilitated regardless of what type of behavior they represent, honest or deceptive. This is because activation of a stereotype or schema facilitates the processing of schema-consistent information, thus reducing associated cognitive load (Macrae et al., 1994; Macrae and Bodenhausen, 2000). On the other hand, the introduction of external cues (EC) and the reliance of subjects on these cues leads to higher cognitive costs of responding deceptively compared to responding honestly because of the necessity to negate truth. We hypothesize that the cognitive costs of deception in predictable social interaction are larger when participants rely on explicit, EC (lie/truth before each question) compared to the same responses in the same context based on activated prior knowledge (without EC). In order to test this hypothesis, we have developed a new experimental task which introduces social context to responses to questions.

## Materials and Methods

### Task

For the purpose of studying deception in the context of social interactions, we have developed the Speed-Dating Task (SDT). SDT is based on a real social event in which participants engage in short conversations, after which they decide if they wanted to meet their speed-date for a real date. After the event is completed and there is a match between participants, the organizer shares phone numbers to the matched pair.

SDT is a variation of SLT, which introduces social context when responding to questions. SDT takes advantage of the general tendency to think about others in terms of categories (Macrae and Bodenhausen, 2000), which in most cases allows for accurate adjustment of behavior.

The questions in SDT are grouped into topics. In the current version of SDT, available topics include religion and “personal philosophy of life” (*Weltanschauung*). A date in SDT refers to a set of questions referring to the same topic, asked in blocks. The change of the date is indicated by an appropriate message on the screen. After each response, feedback is presented on the screen as a smiley or frownie. Smiley indicates that the subject’s response is consistent with the current date’s attitude, while the frownie indicates inconsistency. Each date has a fixed set of response-dependent feedback messages that are contingent with the stereotypical attitude toward the discussed topic. Questions related to each topic are asked by two dates, which represent opposite attitudes (e.g., for religion—atheist and deeply believing catholic). If the subject responded in the same way to the same question when interacting with both dates, he/she would receive positive feedback one time and negative the other time. However, if the subject adapts responses to the attitude of each date, one deceptive and one honest response will be produced. This feature of SDT guarantees that regardless of the true attitude of the subject both honest and dishonest responses will be emitted, assuming that the subject will be asked to adapt his/her responses to each date. Moreover, just as in SLT, each question will provide within-subject control for itself without the need for any explicit cue before each question. However, the above feature is conditional on the presence of clear subject’s attitude toward the matter the question is referring to.

The questions related to each stereotype (atheist, catholic, liberal, conservative) were selected on the basis of a separate web-based questionnaire study. In the questionnaire, the participants were instructed that their goal will be to fill the remaining responses in a questionnaire for another person who managed to fill in only the first four questions. The questions filled by another person are called “diagnostic questions” because they strongly suggested a particular stereotype. There were two response options for each question: yes and no. It was assumed that if a stereotype is activated consistently, the responses to questions will be consistent between participants. The same set of questions was asked twice, preceded with opposite responses to the same diagnostic questions. Two criteria were used for inclusion of the questions in the main task: (1) consistency of predictions between participants and (2) high probability of opposite responses given the activation of different stereotypes. This procedure guaranteed that the same set of questions followed by opposite feedbacks would lead to activation of opposite stereotypes.

**FIGURE 1:**
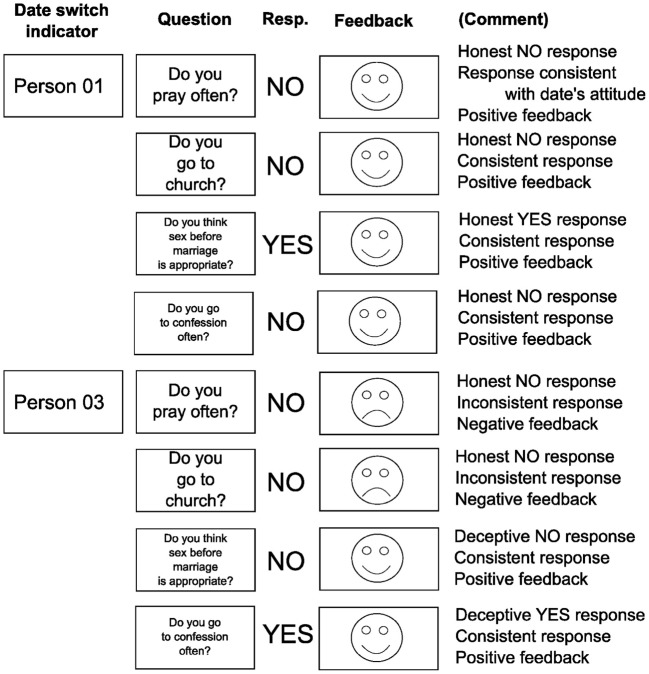
**Speed-Dating Task.** Two representative questions and feedback messages for two different dates. The subject participating in the SDT is an atheist, not participating in any kind of religion. Person 01 is also an atheist with identical attitudes, so every honest response results in positive feedback. However, Person 03 is a faithful catholic, so the feedback following honest responses (questions 1 and 2) is negative, indicating inconsistency. Based on the feedbacks the subject can adjust his responses so that he appears similar to the date (questions 3 and 4). The questions are presented serially.

Example dates are presented in Figure 1. A maximum of 20 questions could be asked for each date. Since the subjects engaged in four dates in this version of SDT, a total maximum of 80 questions were asked during the procedure.

### Participants

Thirty-nine persons participated in the study (20 males). They were pseudo-randomly divided into two groups depending on whether their responses were affected by internal cues (IC) or EC. The randomization ensured that the number of males was equal in both IC and EC groups. The subjects were recruited through a Facebook group related to cognitive neuroscience. Participants received financial compensation for participation (100 zł, ∼25 Euro).

### Procedure

The present study was designed as an fMRI experiment. However, the fMRI data is still being analyzed and the results will be reported elsewhere upon completion. Here we report only behavioral data acquired during the fMRI session.

Before the experiment subjects filled out a written consent for participation in the study and a safety questionnaire for MRI. The study was approved by the University of Social Sciences and Humanities ethics committee. Before the experiment the subjects filled out an online survey where they responded honestly to statements reflecting the questions later asked during SDT. Three response options were available: agree, disagree, and don’t know. Based on these responses, questions with a “don’t know” answer were removed from the task in both IC and EC groups. Response cues were generated if the subject was assigned to the EC group.

The experiment was performed in a 3T Siemens Trio MRI scanner with 32-channel phased-array head coil. The imaging protocol consisted of localizer, 10-min resting-state recording, SDT and structural imaging. The SDT was performed for approximately 19 min. However, the exact duration varied depending on the response pace of the participant. The subjects viewed the stimuli on a 29” LCD monitor standing in the back of the scanner. The LCD was seen through a mirror system mounted on the coil. The subjects responded with two NeuroNordicLab ResponseGrips held in both hands. The responses were made with thumbs. The right thumb corresponded to a YES answer and the left to NO. The cue presenting the side which corresponds to a particular response was always presented with the question, on the bottom part of the screen. Stimulus delivery and behavioral response recording was controlled by Presentation^®^ software (version 17.2).

### Experimental Manipulations

The core manipulation of the study was achieved by randomized group assignment of the subjects. The first group, called internally cued (IC), was instructed that the goal of the SDT is to convince all dates to a real date. The best way to achieve this goal is to adapt the responses to give the impression of similarity. Thus, the IC group had to infer the attitude of each date based on feedbacks and respond on the basis of the activated stereotype. Each question was preceded with an “A” cue, reminding subjects to adapt. The instruction for this group was as follows (direct translation from Polish):

*You are participating in a study about adaptation in social interactions. During the task you will engage in so-called speed-dates with four different persons. The goal of speed-dating is to convince the other person to a real date. Your goal will be to convince* ***all*** *your dates to have a real date with you. Each date will ask questions related to various topics. You will answer YES (right button) or NO (left button). Soon after your response, feedback will be displayed on the screen indicating whether your response was consistent with the current date’s attitude. A smiley will indicate consistency, a frownie—inconsistency. Your job will be to create the impression that you have an identical attitude towards the discussed topic for each date.* ***The letter “A” will be displayed before each question, reminding you to adapt your responses***. *Please respond as fast as possible—the time to respond is limited.*

The second, externally cued (EC) group obtained additional instruction that they will receive aid by having an appropriate cue displayed on the screen before and during the question. Cueing was achieved by replacing the bolded sentence in instruction for the IC group with:

We will help you with that task. Before each question you will see a cue in form of a letter “T”(in green) or “L” (in red). A green letter “T” indicates that an honest response for the following question will be consistent with current date’s attitude. The letter “L” indicates that a deceptive response will be consistent with the date’s attitude. Do your best to follow these cues.

If the honest response was consistent with the date’s attitude, “T” cue appeared and both the cue and question were presented in green. When a deceptive response was appropriate, a “L” cue was presented in red, followed by a red-colored question. The color cue was introduced to ensure that participants remembered the presented cue and did not have to store cue information in working memory. Importantly, if the subject followed the presented cues, all responses were consistent with the date’s attitude. Note that despite introducing explicit instructions, the experimental task did not prevent the EC group from responding based on activated stereotype while ignoring the presented cues.

### Data Analysis

In order to the test the hypotheses of the experiment, linear mixed models (LMM) were used for data analysis. The main advantages of LMMs include explicit modeling of subject- and item-specific random effects, thus increasing statistical power over models averaging the data on the first level of analysis (Baayen et al., 2008).

The RTs are approximately log-normally distributed (Rouder, 2005). Therefore, the dependent variable in the model is the log reaction time (log RT). The reported differences in RT will be reported as percentage change.

The LMM included random intercepts for both subjects and items. These allowed us to control for unmeasured individual differences between subjects (reading speed, IQ, working memory capacity etc.), as well as differences between SDT items, including question length. Random slopes for within-subject main effects and interactions were also included in the model. This allowed to further account for individual differences in effects of interest (Kliegl et al., 2010; Jaeger et al., 2011).

Three factors and their interactions were entered as fixed effects. The within-subject factors included response (honest/adaptive) and question repetition (first/second). The between-subject factor was cue type (external/internal). Interactions of all fixed effects were also included in the fixed-effects part of the statistical model.

In order to test the main hypothesis of the experiment, a contrast analysis was performed on parameter estimates of the statistical model. The main hypothesis of the experiment corresponded to the contrast:

The errors in the SDT are defined as failures to adapt to the current date. These errors were calculated separately for both honest and deceptive responses.

All data analysis was performed in R 3.1.2 (R Core Team, 2015) with auxiliary packages. Parameters of Gaussian-exponential distribution were fit with the **retimes** (Massidda, 2013) package. For LMMs, **lme4** (Bates et al., 2015) was used. The **lmerTest** (Kuznetsova et al., 2014) package was used for p-value approximation for LMMs, and the **multcomp** package was used for planned contrasts (Hothorn et al., 2008). The default single-step multiple comparisons correction method implemented in multcomp was used for all exploratory analyses.

## Results

### Errors

Since the number of trials varied between subjects, error rates instead of counts are formally compared. Two types of errors were possible. The first type is related to a deceptive response while an honest one is consistent with the date’s attitude—a *false alarm*. Errors are presented in Table 1. The total number of false alarm errors committed by both groups was 42 (1.8% of all responses). The IC group made slightly fewer false alarms compared to the EC group [2.6% (min/max: 0–8.1%) vs 3.9% (min/max: 0–18.9%), respectively]. However, this difference was not significant according to Welch 2-sample *t*-test [*t*(32.84) = 1.02, *p* = 0.31]. Nearly half of all false alarms (47.6%–20 of 42) were committed during responding to first 3 questions (11 vs 9 for IC and EC groups, respectively). Within the remaining 52.4% (22) false alarms, the majority was committed by the EC group (81.8%–18). It must be noted that during the whole task 25 of 39 subjects have committed no errors, 10 subjects–1 error, 3 subjects–2 errors and the remaining subject has committed 6 false alarm errors.

**TABLE 1:** **Misses and false alarms committed by both groups in the early (questions 1–3) and late (questions 4) stages of dates**.

		**Group**		
**Error type**	**Date stage**	**EC**	**IC**	**Total**
False alarm	early (Q 1–3)	45% (9)	55% (11)	20
	late (Q 4+)	81,8% (18)	18,2% (4)	22
Miss	early (Q 1–3)	19,1% (9)	80,9% (38)	47
	late (Q 4+)	53,1% (26)	46,9% (23)	49

The second type reflects the opposite—an honest response instead of deceptive—a *miss*. The total number of misses for both groups was 96 (4.1% of all responses). The IC group made more misses type errors compared to the EC group [11.1% (2.6–27.3%) vs 5.32% (0–12%)] and the difference was significant [*t*(26.93) = 3.05, *p* = 0.005]. Approximately half (48.9% –47 of 96) of these errors were committed when responding to first 3 questions asked by dates, with most mistakes made by the IC group (80.9%–38). For the remaining 51.1% (49) questions the number of errors was roughly equal [46.9% (23) and 53.1% (26) for IC and EC groups, respectively].

For a total of 18 items it was not possible to determine the type of response (honest or deceptive). Thus, from the original sample of 2348 RTs, 156 were removed from further analyses (6.6%).

### Reaction Times

#### Raw Data

After exclusion of questions for which the participants had no opinion, the subjects responded on average to 56 questions [SD = 11, range = (31–77)]. Therefore 14 responses per condition were analyzed on average for each subject. Although the number of questions was slightly higher in the EC (57.6) compared to IC group (54.7), the difference was not significant [Welch’s *t*-test: difference 95% CI: (–10.2–4.5), *t*(30.92) = –0.79, *p* = 0.43, Cohen’s *d* = –0.28].

**FIGURE 2:**
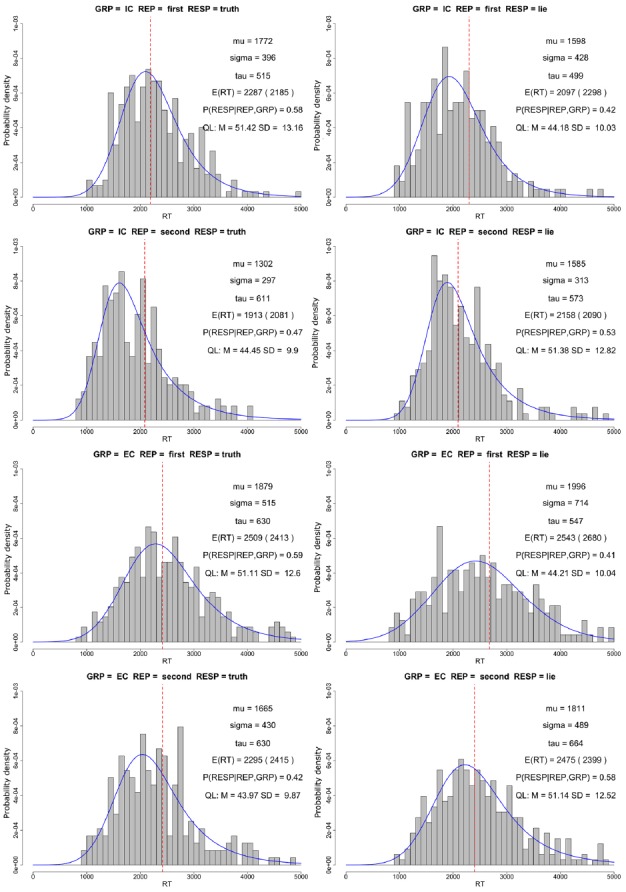
**The histograms represent raw RTs for each group, repetition and response type.** Mu, sigma, and tau values represent parameters of the Gaussian-exponential distribution fit to the raw data (blue line). E(RT) denotes expected value of raw data (mu + tau), and the value in the parentheses represents the expected value based on fixed effects of the statistical model (red vertical line). P(RESP|REP,GRP) denotes the probability of the response presented in each histogram given the repetition and group it belongs to. QL denotes mean (M) and standard deviation (SD) of average question length (number of characters) for the respective conditions.

The histograms of raw data for each condition in the task are presented in Figure 2. For the first presentation of the question, the IC group had higher expected RTs for honest (μ + *τ* = 2287 ms, σ = 396 ms) than deceptive responses (μ + *τ* = 2097 ms, σ = 499 ms). In the EC group, RTs for both conditions were similar (μ + *τ* = 2509 ms, σ = 515 ms for honest and μ + *τ* = 2543 ms, σ = 547 ms for deceptive responses). For the second repetition of the same question, the relationship in IC group was reversed with μ + *τ* = 1913 ms, σ = 297 ms for honest and μ + *τ* = 2158 ms, σ = 313 ms for deceptive responses. In the EC group honest responses were faster (μ + *τ* = 2295 ms, σ = 430 ms) than deceptive responses (μ + *τ* = 2475 ms, σ = 489 ms).

The expected RTs in the EC group are longer in all conditions. Moreover, the distributions of RTs in the EC group seem to be more dispersed (higher σ values) and have heavier right tails (higher *τ* values) compared to the IC group.

The above data suggest some differences between honest and deceptive responses, which is inconsistent with the main hypothesis of this experiment. However, one must consider a very important aspect of the experiment which has profound influence on the measured RTs—the length of presented questions. The length of questions in SDT varied between 27 and 77 characters (including spaces). Assuming average reading speed of 24 ms per letter, due to differences in question length alone 50 × 24 ms = 1200 ms RT difference is expected between the shortest and longest questions. The average question length was not balanced between conditions. This is a side-effect of the structure of SDT. For the first repetition, the length of questions with honest responses was approximately 7 characters longer than deceptive responses in both IC and EC groups. This difference corresponds to 7 × 24 ms = 168 ms expected RT difference between conditions. For the second repetition of questions, the same difference held, but in the opposite direction—questions with deceptive responses had more characters. These question-specific differences, as well as between-subject differences resulting from a varying number of trials per subject, were taken into account by random effect components of the statistical model.

### Confirmatory Analyses

The total R^2^ value for the fitted model was 0.606, with 0.048 for the fixed effects. The distribution of residuals of the model deviated from normal even after log-transformation of the dependent variable, but this behavior was expected. Plotting residual vs predicted values did not show any significant relationship (Pearson’s ρ = 0.05).

**FIGURE 3:**
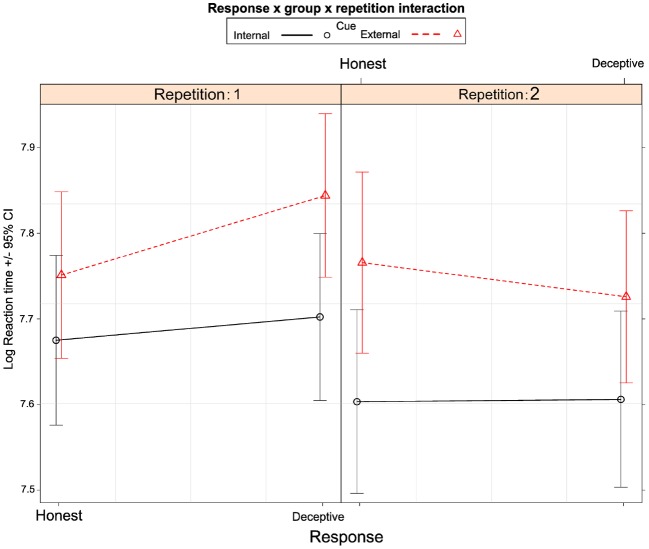
**Least-squares estimates of expected RT values with 95% confidence intervals for all conditions**.

The coefficients along with standard errors and confidence intervals are presented in Table 2. The expected values and confidence intervals based on the fitted model are presented for all conditions in Figure 3.

**TABLE 2:** **Linear mixed model coefficients (non-standardized), standard errors (SE), degrees of freedom (df), *t*-statistic value of a test comparing the parameter estimate to null distribution (t) and the *p*-value testing significance of the difference from 0 (p)**.

**Term**	**Estimate**	**SE**	**df**	***t***	***p***
Intercept	7.675	0.05	62.34	152.025	~ 0
RESP : Deceptive	0.027	0.025	40.06	1.105	0.276
REP : Second	–0.072	0.032	51.84	–2.231	0.03
CUE : External	0.076	0.058	36.75	1.302	0.201
RESP: Deceptive × REP: Second	–0.024	0.032	39.16	–0.767	0.448
RESP: Deceptive × CUE: External	0.066	0.031	36.05	2.118	0.041
REP: Second × CUE: External	0.086	0.038	36.52	2.252	0.03
RESP: Deceptive × REP: Second × CUE: External	–0.108	0.039	32.18	–2.757	0.01

The Intercept term represents expected log RT for honest responses to first repetition of questions in the IC group.

We have found no significant RT differences between deceptive and honest responses in both IC [1.5%, 95% CI: (–2.8–5.77%), *p* = 0.68] and EC groups [2.6%, 95% CI: (–1.49–6.78%), *p* = 0.28]. Contrast analysis showed that in EC group the difference between deceptive and honest responses in terms of RTs was 1.15% [95% CI: (–3.8–6.1%)] larger compared to IC group. This result could not be interpreted as significantly different from 0 (*z* = 0.45, *p* = 0.65). Therefore, we did not find statistical support for our primary hypothesis.

### Exploratory Analyses

Figure 2 indicates that the hypothesized effect might be present only for the first repetition of questions. Thus, we performed an additional contrast analysis testing the main hypothesis only for the first repetition of the question. For the first repetition of the questions, IC group showed no significant difference between deceptive and honest responses [2.7%, 95% CI: (–3.4–8.8%), *p* = 0.7]. RTs in EC group were 9.27% [95% CI: (3.4–15.14%), *p* = 0.0003] higher for deceptive compared to honest responses. The difference between deceptive and honest responses was greater in the externally cued group compared to the internally cued group by 6.56% [95% CI: (0.5–12.6%)], and this difference may be considered as significant (*z* = 2.12, *p* = 0.03). This result provides a partial confirmation of our primary hypothesis. No significant difference between deceptive and honest responses was found for the second repetition of questions in both groups: 0.2%, 95% CI: (–6–6.55%) *p* = 1 and –3.98%, 95% CI: (–10–2.1%), *p* = 0.35 for IC and EC groups, respectively.

Moreover, Figures 2 and 3 also suggests that the externally cued group was generally slower than the internally cued group, consistent with raw data analysis. However, contrast analysis showed that it is not the case. The EC group had 7.6% [95% CI: (–3.8–19%)] longer RTs compared to the IC group, and this difference could not be considered as significant (*z* = 1.3, *p* = 0.19).

## Discussion

In the current paper we hypothesized that the introduction of EC to deception and truth-telling alters the decision-making process, so that it is most likely that deceptive responding will be associated with higher cognitive load compared to truth telling. We argued that the main reason for this effect is the absence of social context in the decision-making process; schemas activated by social context facilitate responses consistent with it.

In order to test this hypothesis we developed the SDT, which is based on a classic DOD/SLT paradigm, but introduces social context when responding to questions. Most importantly the social context is highly predictable for the participants, which is reflected by relatively low error rates regardless of the type of cue that was presented.

We found partial support for our primary hypothesis. The RT difference between deceptive and honest responses was higher in the externally cued group compared to the internally cued group. This effect was present only for the first presentation of questions and not for the second.

We speculate that the main reason why the effect was found only for the first presentation of the question stems from the presence of a predictable social situation in both the internally and externally cued groups. Although the EC group received cues about what type of response was expected, they were not forced to rely on them in the decision-making process. Instead, the subjects could ignore the EC and base their responses on the schemata activated by feedbacks, in the same way as in the IC group. Thus, subjects could transition to the same decision-making process as the IC group and there was no control over this process. However, this result remains largely speculative. No RT differences were found for the second repetition of the questions, suggesting a change in the decision-making process. The transition of the strategy in the EC group after interactions with the first two dates can be explained by accumulation of task-related knowledge. The subjects realized that the interlocutors are stereotypical and responses consistent with their attitudes are easily predictable, thus reducing cognitive load related to responding deceptively. This effect is consistent with CL2 proposition of Information Manipulation Theory 2 (IMT2)—“*the projected cognitive load of potential problem solutions should determine whether one pursues a discourse path that ends up being truthful or deceptive*” (McCornack et al., 2014). In the case of SDT with both a predictable social situation and EC, the subjects tend to choose a less cognitively demanding decision-making process (schema-based) over a more demanding one (cue-based).

The results from error analysis are consistent with the claim that external cuing might be associated with higher cognitive load. Although the IC group committed the majority of *miss*-type errors (honest response when deceptive was consistent with the date’s attitude), half of these errors were committed when responding to first 3 questions asked by dates. Since the IC group had no knowledge about the interlocutors, they responded honestly. Only after observing negative feedbacks after these responses they could activate appropriate schemata and respond in line with it. On the other hand, EC group had the cues presented from the beginning and could rely on them. For the remaining questions the number of *miss* errors was similar for both groups. For *false alarms* (deceptive responses instead of honest), there is no evidence for between-group difference for first 3 questions, but for the remaining questions the majority of false alarms was committed by EC group.

Given the task structure, one must consider that the errors for both groups might emerge from different processes. In the EC group, both types of errors can be understood as failures to comply with the instruction. This is common for instructed-lying paradigms and can be attributed to *failure of cognitive control*. On the other hand, errors in the IC group are likely to emerge from lack of precise knowledge about the interlocutor (or the relationship between response to a particular question and date’s attitude) and could be interpreted as *failures of theory of mind*. However, these interpretations are speculative and further research is needed to examine the nature of errors in different contexts. The total number of errors in the present study is too small (∼6% of all responses) to allow for detailed analyses.

DOD/SLT paradigms provide no social context or purpose to responses emitted by subjects. Thus, no information can enter the working memory before questions are asked. In order to deceive, one indeed has to retrieve the honest information first and then deny it, which requires additional cognitive resources. This process might reflect only a sample from all possible contexts in which deceptive responses might emerge. Although this model seems to apply to high-stakes forensic context, it seems inaccurate with respect to the most frequent low-stakes everyday life deception (Serota et al., 2010). People tend to stay in predictable and stable environments and very rarely engage in unpredictable social interactions. The context provides cues that might facilitate specific kinds of behaviors, including deceptive ones. This increases the availability of deceptive variants of behavior, reducing the cognitive resources necessary to emit such behaviors. Although truthful information might also be simultaneously activated in the WM, our study shows that it does not interfere with deceptive responding. This is consistent with the claims of IMT2 model (McCornack et al., 2014), which places no sharp boundary between honest and deceptive responses in low-stakes situation.

Although the main interest of deception research are high-stakes lies, we believe that paradigms utilizing low-stakes stakes lies with social component (such as the SDT) can be useful in advancing the knowledge about deception in general. SDT provides a framework of incorporating prior knowledge into the experimental deception tasks. Although we have used simple and well-known categories in the current study as a model of prior knowledge, the same concept could be generalized to other social contexts, in which one person tries to create a specific impression on another. The knowledge incorporated into the tasks can be controlled or measured by the experimenter. At the same time, the most important feature of DOD/SLT paradigms (items providing control for themselves) is maintained.

Prior knowledge might also play an important role in high-stakes situations. Activation of prior knowledge by any kind of social cues can facilitate deceptive responding, even in high-stakes situations (Walczyk et al., 2014). Social features might also interfere with honest responding, producing higher cognitive load associated with some honest responses (Littlefield et al., 2015). Therefore, we believe that incorporation and modeling of prior knowledge into experimental deception tasks will allow to further understand the complexity of deception.

### Study Limitations

The current study has important implications; however, limitations also need to be considered. First, the study is severely underpowered. The study had a very limited number of trials per participant and relatively small number of participants per group. Second, the experimental manipulations employed in the study were imperfect. The subjects in the EC group could use both schemata and cue-based decision-making process, which might be the primary reason why the effects were relatively weak and present only for the first presentation of the question. Third, the differences between conditions of interest were confounded by aspects of the items. Although the influence of the confounders can be controlled at statistical level, ideally they should be controlled at the level of experimental procedure. The first, second, and third limitation lead to relatively small effects and large confidence intervals for studied groups.

SDT also falls short in terms of ecological validity. Although effort has been put into modeling the processes present in real-life social interactions (incorporation of prior knowledge and predictability of social situation), the context of the interactions is simplified and one cannot conclude that the same principles would apply to real social interactions. However, we believe that the introduction of simplified social context is an important advance in laboratory-based deception studies and should be pursued further.

## Author Contributions

MF designed the experimental procedure, conducted the experiment, analyzed the data and wrote the manuscript. JS designed the experimental procedure, conducted the experiment and wrote the manuscript. JB conducted the experiment. IS, AG and EN helped design the study, helped with data interpretation and provided critical assessment of the manuscript.

### Conflict of Interest Statement

The authors declare that the research was conducted in the absence of any commercial or financial relationships that could be construed as a potential conflict of interest. The reviewer, Steven A. Mccornack, and handling Editor declared a current collaboration and the handling Editor states that the process nevertheless met the standards of a fair and objective review.
